# Zernike Moment Based Classification of Cosmic Ray Candidate Hits from CMOS Sensors

**DOI:** 10.3390/s21227718

**Published:** 2021-11-19

**Authors:** Olaf Bar, Łukasz Bibrzycki, Michał Niedźwiecki, Marcin Piekarczyk, Krzysztof Rzecki, Tomasz Sośnicki, Sławomir Stuglik, Michał Frontczak, Piotr Homola, David E. Alvarez-Castillo, Thomas Andersen, Arman Tursunov

**Affiliations:** 1 Institute of Computer Science, Pedagogical University of Krakow, 30-084 Kraków, Poland; olaf.bar@up.krakow.pl (O.B.); lukasz.bibrzycki@up.krakow.pl (Ł.B.); marcin.piekarczyk@up.krakow.pl (M.P.); michal.frontczak1@up.krakow.pl (M.F.); 2 Department of Computer Science, Cracow University of Technology, 31-155 Kraków, Poland; 3 Department of Biocybernetics and Biomedical Engineering, AGH University of Science and Technology, 30-059 Kraków, Poland; krz@agh.edu.pl (K.R.); sosnicki@agh.edu.pl (T.S.); 4 Institute of Nuclear Physics, Polish Academy of Sciences, 31-342 Kraków, Poland; slawomir.stuglik@ifj.edu.pl (S.S.); piotr.homola@ifj.edu.pl (P.H.); david.alvarez@ifj.edu.pl (D.E.A.-C.); 5 NSCIR, Thornbury, ON N0H2P0, Canada; tandersen@nscir.ca; 6 Institute of Physics, Silesian University in Opava, Bezručovo nám 13, 74601 Opava, Czech Republic; arman.tursunov@physics.slu.cz

**Keywords:** CMOS sensors, feature-based classification, Zernike moments, machine learning, computer vision

## Abstract

Reliable tools for artefact rejection and signal classification are a must for cosmic ray detection experiments based on CMOS technology. In this paper, we analyse the fitness of several feature-based statistical classifiers for the classification of particle candidate hits in four categories: spots, tracks, worms and artefacts. We use Zernike moments of the image function as feature carriers and propose a preprocessing and denoising scheme to make the feature extraction more efficient. As opposed to convolution neural network classifiers, the feature-based classifiers allow for establishing a connection between features and geometrical properties of candidate hits. Apart from basic classifiers we also consider their ensemble extensions and find these extensions generally better performing than basic versions, with an average recognition accuracy of 88%.

## 1. Introduction

Cosmic rays are actively studied by astrophysicists, due to their as-yet-unknown unknown origin and enormous peak energies. They also have important implications for radiation safety [1], the operation of electronic devices working both on Earth and in space [2,3] and even earthquake prediction [4,5,6]. Studying cosmic rays by using a worldwide network of mobile devices as an extremely distributed radiation detector was proposed by several research groups [7,8,9] and can be treated as an example of the citizen science paradigm. Practical application of this paradigm requires overcoming several obstacles like the identification and rejection of artefacts: i.e., images that cannot be attributed to a particle’s passage through the sensor. The emergence of artefacts may result from deficiencies of the registration procedure, the finite rejection capability of the online trigger algorithm, or the fact that some users cheat.

In this paper, we describe a method of signal classification for cosmic ray images, called hits, obtained from CMOS sensors mounted in smartphones equipped with the specialized CREDO Detector application [10]. Unlike other solutions discussed in the literature [11,12,13] where Convolutional Neural Networks were used for the online or offline trigger and offline signal classification, respectively, we instead use a feature-based solution. As feature carriers we use Zernike moments which are known to faithfully map the complex geometrical properties of images albeit at the cost of substantial noise sensitivity [14,15,16,17].

We have performed feature-based classification using a series of statistical classifiers based on supervised learning, such as *k*-nearest neighbour, decision trees, Gaussian Naive Bayes, linear and quadratic discriminant analysis, logistic regression, support vector machine, and neural network [18,19,20]. For some of the above classifiers we also tested their ensemble versions [21,22,23]. The search for optimal settings for each classifier was implemented as a two-phase process. The first phase was concerned with identifying the optimal set of hyperparameters for the classifier model under analysis. The second phase involved a more precise evaluation of the performance of a given classifier by assessing its generalization potential and evaluating statistical uncertainty.

Feature-based classifiers, even though not entirely explainable, have the advantage that they make it possible to establish a connection between the components of feature vectors and image properties observable with the naked eye. In what follows, we focus on the morphological properties of particle tracks rather than their physical interpretation. Such interpretations can be found in the literature [13,24] where e.g., long thin tracks were associated with muons while wavy tracks (worms) with Compton recoil electrons. However, unambiguous mapping between track shapes and radiation types is difficult for commodity smartphones. It requires detailed studies of radiation propagation in a sensor of a given geometry and to the authors’ best knowledge had not been performed so far for the representative set of the devices available on the market. Such an analysis is complicated in many respects. From the theoretical point of view, to be able to assign a specific shape to a given particle (e.g., muon, electron or proton) it is necessary to do tests and investigations under laboratory conditions during which a device with a CCD/CMOS matrix would be irradiated with only one particle species. Such tests could show how a given particle looks like and if it always has the same shape, e.g., if a muon is always a line. There are quite a few publications on the analysis and identification of particles based on traces [25] with a catalogue of shape names like spots, track and worms, that we adopt here.

From the practical point of view, the problem is how to recognize if a trace is caused by a genuine cosmic ray rather than by a local radiation event without the knowledge of energy or the environmental conditions (local sources) in which the application was used. Moreover, very often a trace cannot be assigned to a specific category - it has an intermediate shape between two categories.

**Figure 1:**
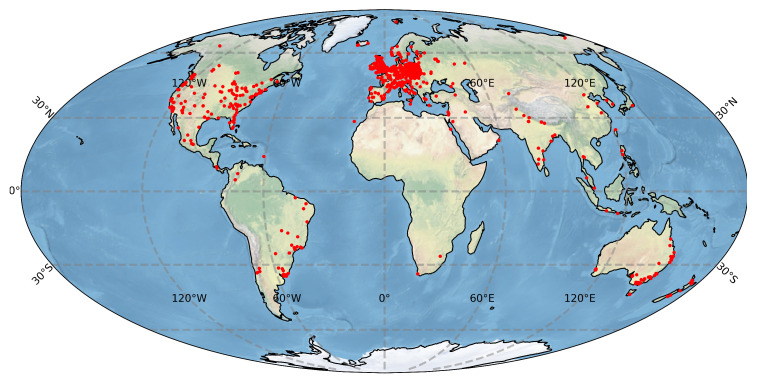
Approximate locations of phone-based detectors registered by the CREDO project (as of the beginning of 2021).

Notwithstanding the large number of applications, like in nuclear and particle physics [26], astroparticle physics [27,28] or medical imaging [29], all semiconductor detectors are based on the same principle of collecting and analysing the charge liberated by incident radiation in the depletion region. Noteworthy, these same physical processes occur in CMOS sensors in domestic electronic devices like video recorders or the digital cameras used by mobile phones. Combined, smartphone ubiquity and their internet connectivity made them an ideal framework for creating a global network of radiation detectors coupled to central data storage. This idea underpinned several particle detection initiatives like CRAYFIS [7,30], DECO [8,13] and CREDO [9,10]. Our analysis is based on the CREDO detection data set, as currently, this is the largest publicly available data set of particle images obtained with mobile phones. The span of the CREDO device worldwide infrastructure is shown in Figure 1. It is worth noting that currently the total number of registering devices is over 10 thousand and is on the rise.

Such a large number of detections requires an effective and efficient tool to recognize various types of hits and filter out artefacts. To give an idea of the scale of the CREDO experiment and its associated data set of images, in Table 1 we show key metrics drawn from the CREDO database as of September 2021.

## 2. Classification of Candidate Hits: Strategy Overview

### 2.1. Overall Problem Formulation

In the paper, we discuss the problem of image classification of candidate cosmic ray hits represented as 3-channel RGB arrays. Images have been divided into four classes: spots, tracks, worms, and artefacts, as described in the next subsection.

On the formal side, the classification task undertaken in this paper can be defined as the search for some mapping *A* described by Equation (1):

(1) A:D⟶I,

where: *A* denotes the function describing the classification process, *D* denotes the input data in the form of an array representing an RGB image and *I* denotes the set of classes to be the result of recognition. The mapping *A* is realized in practice as a composition of three transformations according to Equation (2)

(2) $$A=D_{f}\cdot M_{f}\cdot R_{F},$$

where: $R_{F}$ denotes feature reception, $M_{f}$ denotes the process of computing the values of the membership function and $D_{f}$ denotes the process of deciding the final classification.

The reception of features is understood here as the conversion of d∈D objects into points of a certain space *X*. This transformation is carried out by selecting and then measuring the features of the objects represented by the input data, i.e., the implementation of the mapping defined by Equation (3)

(3) $$R_{f}:D⟶X,$$

The mapping $M_{f}$ is about a measure of the similarity of the unknown d∈D object to individual classes $D^{i}$ and indexed with the numbers i∈I. Assuming the choice of *L* features for object description, this type of mapping can be described by Equation (4)

(4) $$M_{f}:X⟶R^{L},$$

For the statistical classifiers considered in this paper, the inference of the form of the mapping *A* is basically performed in two steps as per Equation (5)

(5) $$A=C\cdot R_{F},$$

where *C* denotes a complex classifier algorithm that integrates $D_{f}$ and $M_{f}$ processes in its structure. In this reformulation, the feature determination process is separated from the modelling of the recognition function and decision making which is performed by the classifier e.g., SVM, kNN or RF. On the other hand, in the model implemented in the deep learning paradigm, even this entire process along with feature reception is performed in an integrated process by the classifier itself e.g., CNN or RNN. Therefore, the complex process of data flow and transformations were undertaken to achieve the end-to-end statistical classification considered in this paper can be defined by the following separated steps:

Step 1. Data augmentation (Section 3.1),
Step 2. Data preprocessing (Section 3.2),
Step 3. Two-phase model evaluation (Section 4 and Section 5).

The dataset preprocessing usually is performed at the beginning of the analysis. In our study, we have had at our disposal event classes of very disproportional sizes, which we split into test and validation sets. In addition, data augmentation had been performed before data preprocessing. A more detailed description of computation experiments with flowcharts describing these experiments are given in Section 4.

### 2.2. Annotated Dataset

In this study, we use the data set originally introduced in [12]. Recall that this set was annotated so that each image was assigned to one of the four classes: spots, tracks, worms and artefacts. The multiplicities of each class are indicated in Table 2.

**Figure 2:**
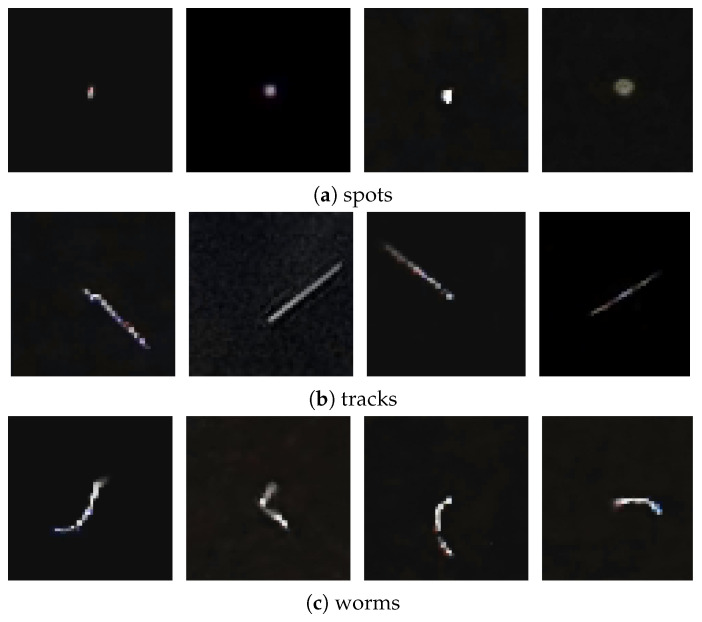
Four representatives of spots (**a**), tracks (**b**) and worms (**c**).

**Figure 3:**
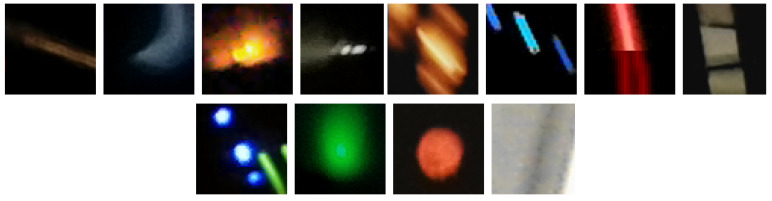
Example of bad detections in an application classified as artefacts. Artefacts are usually the result of not covering the camera, being too close to a light source or user interference in creating detections.

To have a better idea of the three main types of images appearing in the set of detections, in Figure 2 we show examples of spots, tracks and worms. In addition, Figure 3 shows artefacts, i.e., images that do not correspond to particle’s passage through the detector.

Compared to the basic classes of detection images (i.e., spots, tracks, and worms), the artefacts exhibit high brightness and large morphological diversity, making it difficult to classify them. Therefore this class of images was the most numerous in the data set.

### 2.3. Classification Methods

The classification methods that have been investigated for their usefulness in recognizing particle tracks in images are briefly discussed below. The three-letter acronyms denote the types of classifiers and are then used in Section 5 to describe the results. The implementations of the classifiers are provided in the scikit-learn software library [31] were used during the computations. The parameters of the models tested that are not listed below were set to the default values.

- DTC – The decision tree classifier with the criterion parameter tested for values: gini and entropy, and the splitter parameter tested for values: best and random.
- GNB – The Gaussian Naive Bayes (GaussianNB) based classifier with no parameters to be optimized.
- *k*NN – *k* nearest neighbors classifier with the n_neighbors tested in range from 1 to 10 and distance metric parameter for values from set: {euclidean, manhattan, chebyshev and minkowski}.
- LDA – The Linear Discriminant Analysis based classifier with the solver parameter tested for values: {svd, lsqr, eigen}, and the shrinkage parameter tested for values: None and auto.
- LRC – The Logistic Regression (aka logit, MaxEnt) based classifier with the penalty parameter tested for values from {l1, l2, elasticnet, none, the max_iter equal to 100,000, and the solver parameter tested for values from set: {*newton-cg, lbfgs, liblinear, sag, saga*}.
- LSV – The Linear Support Vector Classification - SVM based classifier with the *C* parameter tested in range from 10 to 50, the max_iter equal to 10,000, and the class_weight parameter equal to 1 or balanced as inversely proportional to class frequencies.
- MLP – The Multi-layer Perceptron based classifier with activation parameter tested for values from set {identity, logistic, tanh, relu}, the solver parameter tested for values from set: {lbfgs, sgd, adam}, the max_iter equal to 100,000, and the number of hidden layers equal to 2 or 3 with the hidden_layer_sizes tested in range from 100 to 200 with step 20.
- NSV – The ν-Support Vector Classification based classifier with the radial basis function kernel - RBF, the ν parameter tested in range from 0.01 to 0.10 with step 0.01 and the gamma parameter tested in range from 0.1 to 10.0 with step equal to 0.5.
- QDA – The Quadratic Discriminant Analysis based classifier with the parameter reg_param to regularize the per-class covariance estimates tested in range from 0.0001 to 0.1000 with step 0.0001.
- SGD – The Linear SVM based classifier with SGD training was tested with parameter loss from set: {hinge, log, modified_huber, squared_hinge, perceptron, squared_loss, huber, epsilon_insensitive, squared_epsilon_insensitive}, the penalty parameter equal to l1, alpha parameter values tested from 0.00001 to 0.00010 with step 0.00001, and the max_iter equal to 100,000.
- SVC – The C-Support Vector Classifier with radial basis function - RBF as a kernel, the *C* regularization parameter tested in the range from 500 to 1000 with step 20, and gamma parameter for the RBF kernel tested for values in the range from 0.01 to 0.20 with step 0.01.

The difficulty of solving the classification problem addressed in this paper stems directly from the complexity of the multi-class analysis itself and the limited size of the reference image dataset. Furthermore, the research task is complicated by the fact that at least two of the identified classes, i.e., tracks and worms, appear to be difficult to separate due to their similar visual morphology. An extensive analysis of the problem defined in such terms encouraged us to investigate how ensemble strategies perform under such constraints. Therefore, in addition to classifiers based on basic models, we also evaluated the recognition performance of selected metaheuristics that generate ensemble models. The types of classifiers considered and their parameters under optimization are presented below:

- ETC—An extra-trees classifier is the meta estimator for decision trees with the parameter n_estimators tested in a range from 10 to 100 with step equal to 10, the criterion parameter set either to gini or entropy values, the max_features tested in range from 1 to 10 with step 1 and the bootstrap parameter set to True or False.
- GBC—The Gradient Boosting for classification with the n_estimators parameter tested in the range from 10 to 90 with step equal to 20, the max_depth of the individual regression estimators in range from 1 to 10 with step equal to 2, the learning_rate parameter in range from 0.1 to 0.9 with step equal to 0.2.
- RFC—A random forest classifier, a meta estimator for decision trees with the same parameters tested as for the extra-trees classifier described above.
- BAG—A Bagging classifier that fits base classifier on random subsets to further aggregate their joint predictions was tested for the max_samples parameter in the range from 0.1 to 0.9 with step 0.1, and the n_estimators set to 100.
- OVO—The classifier that implements one-vs.-one multiclass strategy with no parameters being optimized.
- OVR—The classifier that implements the one-vs.-rest multiclass strategy with no parameters being optimized.
- VOT—The hard voting based classifier with weights determining the impact of individual classifiers on the final class assignment.

### 2.4. Zernike Moments as Feature Carriers

Recent analyses of data acquired by the CREDO experiment employed various CNN architectures to detect potentially relevant signals [12] and classify them [32]. Here, rather than CNN based classifiers, we discuss an approach based on the classical statistical learning classifiers implemented in the sci-kit-learn library [31]. The Zernike moments were chosen as feature carriers [14,15,16,17] and the Mahotas [33] library was used to compute them. We chose to employ Zernike moments up to order 8. Numerical experiments showed that the use of higher-order moments did not improve the classification results. Zernike moments are defined on a unit disc in terms a complete and orthonormal set of functions $V_{nm}\left(x,y\right)=V_{nm}\left(r,\theta \right)=R_{nm}\left(r\right)e^{im\theta }$, where *r* is the distance from the disc center and θ is the polar angle defined with respect to the x−axis. The radial polynomials $R_{nm}$ are defined as

(6) $$R_{nm}\left(r\right)=\sum_{j=0}^{\frac{n-|m|}{2}}{\left(-1\right)}^{j}\frac{(n-j)!}{j!\left(\frac{n+|m|}{2}-j\right)!\left(\frac{n-|m|}{2}-j\right)!}r^{n-2j}$$

where *n* index is non-negative and *m* index is bound by conditions that the difference n−|m| has even value and |m|≤n. The basis functions are orthonormal on the unit disc so that

(7) $$\int_{0}^{2\pi }d\theta \int_{0}^{1}drV_{nm}^{*}\left(r,\theta \right)V_{pq}\left(r,\theta \right)=\frac{\pi }{n+1}\delta_{np}\delta_{pq}.$$

Zernike moments are thus projections of the f(x,y)=f(r,θ) image function on the above defined basis functions

(8) $$A_{nm}=\frac{n+1}{\pi }\int \int_{x^{2}+y^{2}\leq 1}f\left(x,y\right)V_{nm}^{*}\left(x,y\right)dxdy.$$

For the computation of Zernike moments of the discrete image function, the integrals are replaced by the summation over the pixels

(9) $$A_{nm}=\frac{n+1}{\pi }\sum_{x}\sum_{y}f\left(x,y\right)V_{nm}^{*}\left(x,y\right),\;x^{2}+y^{2}\leq 1,$$

with *x* and *y* indexing the columns and rows of the image array. A straightforward consequence of the Zernike moments definition is the rotational invariance of their moduli. This considerably, but not entirely, reduces the need for feature augmentation. Some augmentation is still needed to account for the discrete nature of the images which breaks the exact rotational invariance and induces some error [16,34,35]. Still, 10th order Zernike moments are efficient feature extractors for the face expression recognition even in the presence of noise [36]. Given the relative simplicity of the cosmic ray hits, we use 8th order Zernike moments as feature extractors.

**Figure 4:**
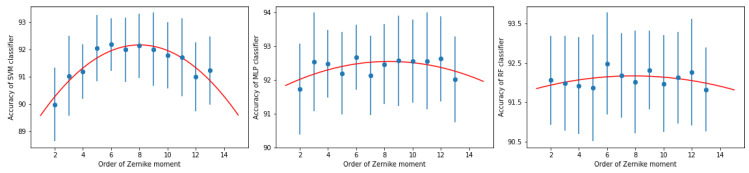
Dependence of the total accuracy on the maximum order of the Zernike moment for SVM (**left column**), MLP (**middle column**) and RF (**right column**). Error bars represent the standard deviation.

To verify this choice we examined the accuracy dependence on the maximum Zernike moment order. We performed this study for three classifiers, i.e., SVM, MLP and RF. The results of this comparison are shown in Figure 4 where we average classification accuracies and their standard deviations are shown. Additionally, we overlaid these results with lowest order polynomial (which turned out to be 2) able to fit them.

### 2.5. Efficiency of Zernike Moment Based Features

**Figure 5:**
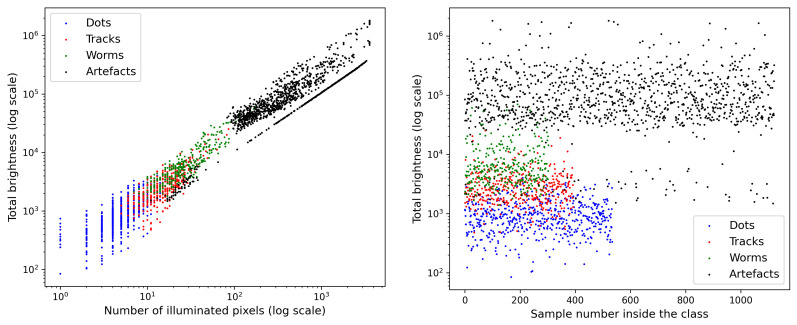
Illustration of the separation of different signal categories with respect to the cumulative brightness and number of illuminated points: (**left**) brightness and number of pixels plot, (**right**) brightness plot.

The comparison of the total brightness of the samples (Figure 2 and Figure 3), shows that this parameter obviously divides the samples into 3 classes: 1—spots, 2—tracks+worms, 3—artefacts, which is demonstrated in Figure 5. The luminosity based separation of artefacts vs. other classes was employed in the discussion of the CNN based trigger in [12].

**Figure 6:**
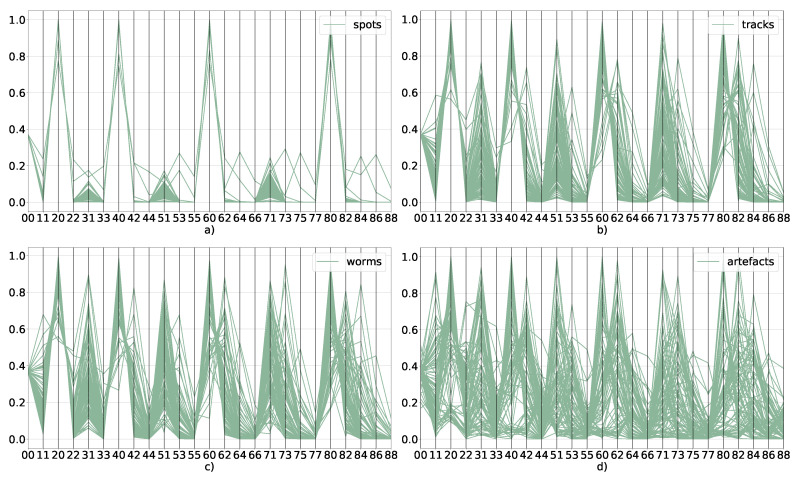
Parallel coordinate plots showing normalized values of Zernike moments for 95 examples of spots (**a**), tracks (**b**), worms (**c**) and artefacts (**d**). Numbers on the horizontal axes exhibit the *n* and *m* indices as per Equation (9).

This qualitative division is also supported by the properties of the Zernike moment spectrum. Figure 6 shows parallel coordinate plots for Zernike moments up to n=8,m=8, for spots, worms, tracks, and artefacts. It is clear that the sets of moments activated across various classes vary considerably. In particular spots activate fewer moments than the other classes with the artefacts activating almost all moments. It is also evident that the difference between worms and tracks as well as between worms and artefacts is less dramatic, compare e.g., Figure 6b–d. These qualitative properties are reflected in the classification results, which we discuss in what follows. Zernike moments (in Mahotas implementation [33]) are normalised to absolute luminance, so to recover this information, we have added it as an additional feature in the form of average luminance.

Our experiments proved that adding this additional feature improves the results significantly, as expected from Figure 5 and Figure 6. Consequently, the input data vector has a dimension of 25 (24 absolute values of the Zernike moments + 1 average brightness).

## 3. Classifier Input Representation

### 3.1. Data Augmentation

**Figure 7:**
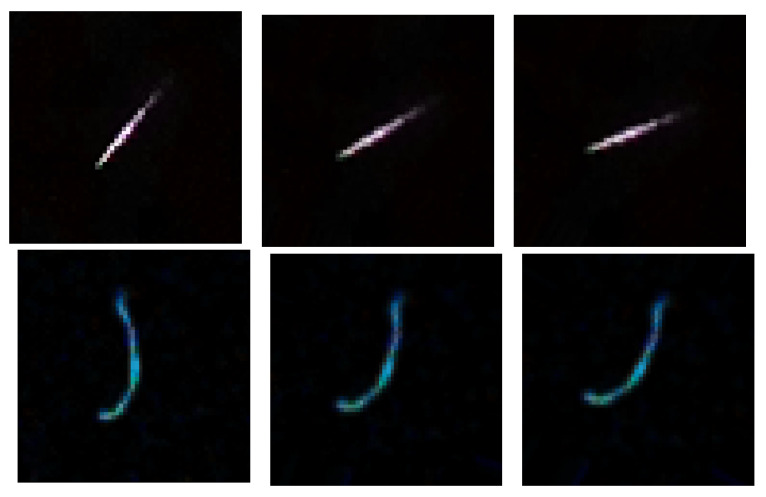
Original track (**upper row**) and worm (**lower row**) and their augmented versions.

A frequently used method to improve machine learning efficiency is the augmentation. It consists in increasing the size of the training set using simple image transformations that do not change their fundamental properties. In the case discussed here, the motivation for the augmentation stems from two facts: the approximate character of the image rotation invariance and the apparent imbalance of the annotated dataset as shown in Table 2. In continuous space Zernike moments acquire a trivial phase factor under rotation, thus their moduli are rotation invariant. This invariance is broken, however, due to the finite size of the pixelization. To account for that, apart from the moments computed for original images, we also compute and use in training, the moments obtained from images augmented by a random rotation within $\pm 20^{o}$ limits (see Figure 7). It was also verified that other transformations (such as scaling and translations) do not improve the performance of the classifiers. Due to the imbalance in the annotated dataset, the track and worm classes are strongly underrepresented: thus, precisely these two classes were subject to augmentation, with multiplication factors of 6 and 12 respectively. Another reason to exclude spots and artefacts from the augmentation was their de facto rotational invariance. The datasets resulting from the augmentation are summarized in Tables 3 and 4 below.

### 3.2. Data Preprocessing

The raw data of the single hit has been stored as a three-channel RGB images [37] and represented as 60 × 60 × 3 arrays with 8-bit precision per channel. The original images were then subject to the grayscale transformation realized by summing up the luminosities across the channels. Finally, the grayscaled images were denoised by applying the threshold calculated as a linear function of the mean and standard deviation of pixel values as defined in Equations (10) and (11).

(10) $$t=\bar{b}+5\sigma$$

where $\bar{b}$ denotes the mean of brightness and σ is a standard deviation of brightness. Finally, the formula that defines the noise cut-off level reads:

(11) $$\begin{array}{c} threshold=\left\{\begin{array}{c} t\;\;\mathrm{for}\;\;t<100\\ 100\;\;\mathrm{for}\;\;t\geq 100 \end{array}\right. \end{array}$$

**Figure 8:**
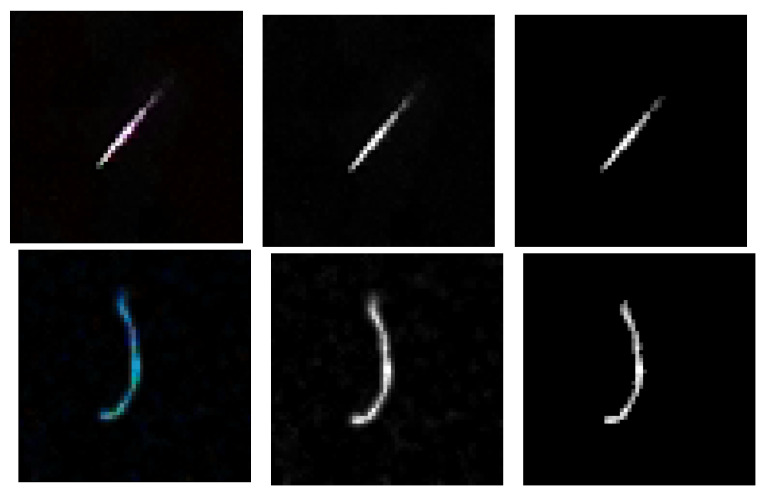
Original track (**upper row**) and worm (**lower row**) and their grayscaled and thresholded versions.

In Figure 8 we show the original track and worm images as well as their grayscaled and thresholded versions.

### 3.3. Feature Extraction

After denoising, for each image the feature vector is obtained by computing the set of Zernike moments absolute values, which are rotation invariant. The length of this vector depends on the maximum degree of Zernike moments taken into consideration - 8th in our analysis. The moduli of Zernike moments are normalized to the integrated brightness, so $z_{00}$ is constant. Therefore we skip this moment and take into account moments $z_{11}$ to $z_{88}$ (24 moments). To recover the information on the image total brightness *b*, we add it as an additional feature. So, the final form of the feature vector reads

(12) $$v=(z_{11},\cdots ,z_{88},b)$$

and consists of 25 components. The final step of data preprocessing was feature scaling which could be performed in several ways: the particular method chosen is detailed in Tables 5 and 6 of Section 5.

## 4. Two-Phase Model Evaluation Scheme

### 4.1. Phase One—Hyperparameter Optimization

**Figure 9:**
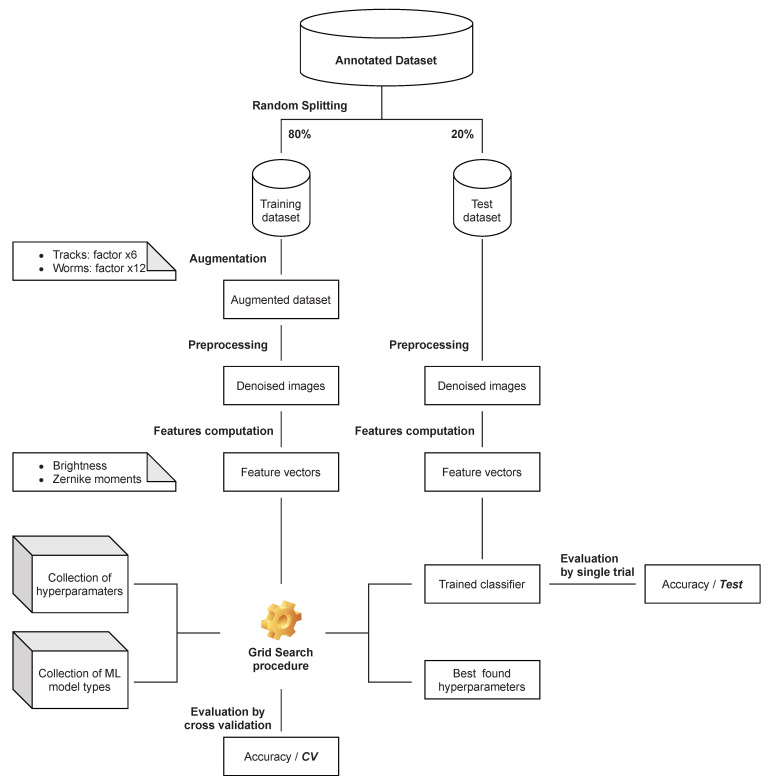
Descriptive scheme for the first phase of computation: parametric optimization of selected classifier models.

The first phase aimed to select the most accurate classification system for the dataset under consideration. This goal was directly realized by searching for the optimal hyperparameters for each type of classifier. The general scheme of the procedure is shown in Figure 9. The lines in the diagram represent the operations being performed and the blocks represent the input and result data/objects.

First, the annotated dataset was divided into a training subset and a test subset in a ratio of 80 to 20. The data was divided in a stratified manner, meaning that the abundances of each class in the two resulting subsets were maintained. The training subset was further augmented to increase the representativeness of the learning data. Only images belonging to the track and worm classes underwent this operation. These signals are characterized by similar morphology, so increasing the information potential of the training subset by increasing the number of variants of these samples is justified. The operation was implemented by applying a random rotation within the ±20 degrees interval as was discussed in Section 3.1. For each sample, additional 6 (tracks) or 12 (worms) new elements were generated. The relevant statistics for augmented data subsets are presented in Table 3. Applying a larger value of the rotation angle is not necessary, nor advisable, since the feature descriptor type used (absolute value of Zernike Moments) is basically rotation invariant. The test subset is not subject to the augmentation process.

At the augmentation stage, all data are still stored as colour images (three channels, RGB) and may contain significant noise. Extraction of the signal itself is performed by converting it to grayscale format, followed by thresholding. The transformation to grayscale is performed by directly summing up the luminance values in all color channels. In this case, we do not use a luminance-preserving conversion mechanism that is suitable for human image perception. The goal is to determine the total energy deposit recorded at each individual pixel in the image frame. The result is a monochrome image that is then subjected to adaptive thresholding with hysteresis (Section 3.2). This operation is intended to eliminate noise and extract the maximum spatially coherent representation of the signal.

The monochromatic image obtained during these transformations is the basis for computing the the feature vector (Section 2.4, Section 2.5 and Section 3.3). For this purpose, a set of features relating to the shape/morphology of the signal (a set of Zernike moments of appropriate order) and, as a complementary feature, a measure of the total energy carried by the signal (brightness) are calculated.

Having a representation in the form of a feature vector, we can proceed to search for an optimal classifier. For this purpose, the well-known Grid Search method is applied, which uses the augmented training subset separated at an earlier stage. The optimization process is performed for the key hyperparameters indicated for each classifier type. As an internal mechanism for evaluating classifier performance, the method uses 5-fold cross-validation. At this stage we use the Grid Search and Cross Validation implementation available in the sci-kit-learn package. The result is an optimal set of hyperparameters, a trained classifier, and classification accuracy (CV). The trained model is then pre-validated by evaluating it against a separate test subset (Test).

### 4.2. Phase Two—Model Statistical Robustness

The second phase of the experiment is to statistically evaluate the performance of the classifier obtained in phase one. In this phase the data set was randomly split into a training set and test set. The train-test split was performed using stratified sampling with proportions of 80 to 20. Next, the training data was augmented (see Section 3.1) and used to train the model. The test set was used for evaluation of the model. The whole process (splitting, training and evaluation) was performed 30 times. The data subset sizes used in the second experiment are collected in Table 4. After completing this phase, we obtain metrics in the form of the average accuracy of classification based on the measurements taken (Mean30) and standard deviation (Std30).

## 5. Experiment Results

In the following subsections, we present the classification results and optimal hyperparameters obtained in the optimization phase as well as their mean accuracies and standard deviations from the evaluation phase for both basic classifiers and ensemble models. All classification computations were performed using the scikit-learn [31] library from the scientific Python ecosystem.

### 5.1. Basic Classifiers

In Table 5 we show results of the experiments obtained for basic classifiers. The first column denotes tested classifiers. The second and third columns, labelled CV and Test, denote the mean accuracy of a given classifier during parameters optimization (cross-validation) and over the test set.

These results in Table 5 show that the best performing methods are the MLP, NSV and SVC. The results obtained in the first (optimization) phase are confirmed by the figures obtained in the second (evaluation) phase. This makes us justifiably confident that our results are statistically robust. In experiments discussed in Section 5.2 the best performing basic classifiers were used as a basis for ensemble models utilizing the voting strategy.

### 5.2. Ensemble Classifiers

In Table 6 we show the results of the optimization and evaluation for various ensemble classifiers. The columns contain information in the same format as in previous tables.

Even though in the optimization phase some accuracy fluctuations across various classifiers can be observed, the validation phase results show that their scores are compatible within one standard deviation.

### 5.3. Classifier Benchmarking

Comparing results in Table 5 and Table 6, we conclude that best performing basic classifiers ie. MLP, NSV and SVC achieve accuracies comparable to those of ensemble classifiers. The three best performing basic classifiers are (1) the Multi-layer Perceptron-based classifier with the relu activation function, the adam solver, and two hidden layers of size respectively 180 and 120, (2) the ν-Support Vector Classification based classifier with the radial basis function kernel - RBF, the ν parameter equal to 0.05 and the gamma parameter equal to 0.1, and (3) the C-Support Vector Classifier with radial basis function - RBF as a kernel, the *C* regularization parameter equal to 700, and the gamma parameter for the RBF kernel equal to 0.08. All of these classifiers’ achieved accuracies over 0.87, calculated during cross-validation, test and the 30-fold procedure of random split, train and test.

**Figure 11:**
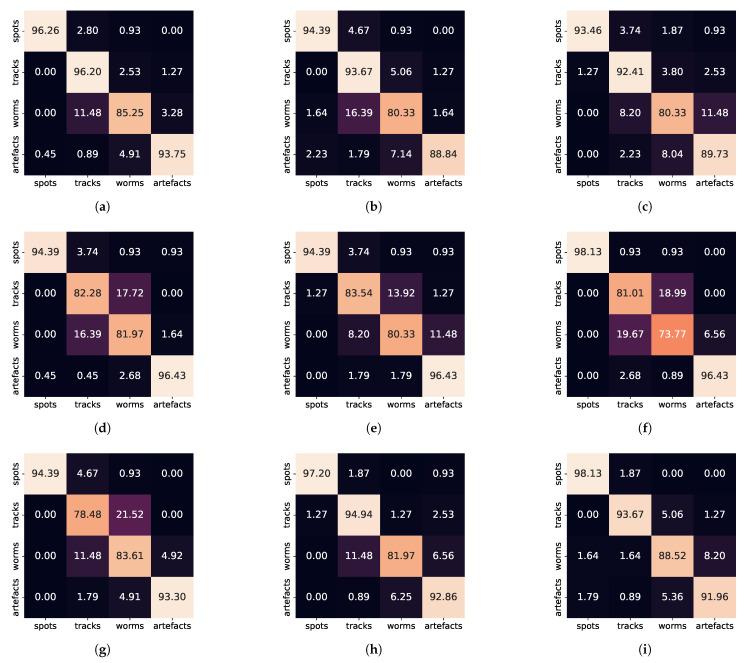
Confusion matrices for best performing simple classifiers: (**a**) MLP, (**b**) νSV, (**c**) SVC and ensemble classifiers: (**d**) ETC, (**e**) GBC, (**f**) RFC, (**g**) bagged SVC, (**h**) SVC with one-vs-one multiclass strategy, (**i**) MLP with one-vs-rest multiclass strategy.

In Figure 11 we show the confusion matrices to illustrate the classification accuracy rate of best-performing classifiers. Not surprisingly all classifiers perform best on the spot class which is morphologically the simplest one. This class is most likely to be confused with the track class. Seemingly, the annotators were not consistent in annotating the slightly elongated spots and annotated them as lines occasionally. The artefact recognition rate was consistently higher than 90%. The worm class recognition rates were generally lower than 90%. This class was usually confused with tracks and to lesser extent with artefacts. The recognition rate of tracks was strongly conditioned by the classifier’s capability to distinguish tracks and worms. Those classifiers which were able to achieve this (such as MLP, νSV, SVC, OVO/SVC and OVR/MLP) performed with an accuracy exceeding 90%.

The analysis and comparison of the accuracy values in columns CV, Test, Mean30, and Std30 for specific classifiers allow one, to some extent, to assess their general properties, e.g., robustness, and the degree of confidence in their ability to generalize performance on an unknown input set. The following simple conclusions seem legitimate with a high degree of confidence:

- CV accuracy significantly higher than Test value indicates overfitting (e.g., KNN and LDA).
- Mean30 accuracy significantly lower than Test value suggests that the classifier parameters were specific to the optimization set (this problem is marginally observable for NSV).
- High Std30 value would suggests that the given classifier is unstable. We do not see such cases in our results, therefore we conclude that all classifiers’ class assignments are fairly robust.

We can generally conclude that most of the best performing classifiers utilize the ensemble approach, achieving accuracies close to 90%.

### 5.4. Ensemble Classifiers vs. CNNs

In the present work, the MLP classifiers with one-vs-rest multiclass strategy provide good recognition capabilities for all four classes of events with small contamination from wrong identification. These results are compared in Table 7 with results from [13,32].

There is a certain improvement in the Track recognition capability with the new approach when compared to [32] without too much deterioration in identifying the Worms. Although, the contamination of worms identified as tracks and vice-versa is reduced. An important difference, however, is that in [32] the threshold scheme was applied thus leaving a significant fraction of events unclassified if they did not pass the probability threshold. The recognition accuracy obtained in the present work seems to be consistently inferior to that obtained by [13]. But again we note that in [13] the class assignment accuracies were computed only for examples passing the threshold defined as at least 80% probability to belong to a given class. Eg. for the spot class the rejection rate was over 50%. Our approach does not admit inconclusive classifications which is reflected in slightly lower overall accuracy. Using the absolute values of Zernike moments, that by definition are the rotationally invariant, reduces the need for strong data augmentation through rotation. It was observed that scaling and translations do not improve the performance of classifiers when used with Zernike moments. Hence, as shown in the present work, the Zernike moments when used with the best-performing ensemble classifiers give very similar results when compared to the CNNs without the need for strong data augmentation.

## 6. Conclusions

In this paper, we have evaluated a series of statistical classifiers, both basic ones as well as ensemble meta-estimators. After optimizing each classifiers hyperparameters, the most successful of the trained classifiers performed with an accuracy close to 89%. Classifiers that score high in classification accuracy are mostly ensemble models. This is not surprising, as this type of meta estimator has a great potential to tackle the tasks that require dealing with hard-to-distinguish object categories. An interesting observation is that high performance was also obtained for the ensemble architecture based on a set of heterogeneous classifiers. This is the case for an ensemble model using a voting strategy involving a group of MLP, NSV and SVC classifiers. From a research point of view, it will also be interesting to verify whether such heterogeneous ensemble classifiers as proposed in this work behave similarly to homogeneous classifiers based on boosting or bagging in terms of the shape of decision boundaries between classes.

Not surprisingly the classifiers perform best at recognising the spot and artefact classes while having significantly worse precision and recall values for tracks and worms. This, however, does not imply the failure of the classification method itself but rather is a consequence of the underlying lack of annotators’ unanimous agreement.

Zernike moments proved their usefulness as feature extractors albeit with the necessity to remove the noise with carefully developed preprocessing methods. Therefore the solutions described in this paper can be applied for the real time analysis of the data stream received from CREDO Detectors.

The application of models analyzed in this paper will allow for a more comprehensive analyses of the full CREDO dataset from the point of view of the morphology of the registered particle trajectories. This will allow pioneering on large scale analyses of the CREDO dataset, validating the actual effectiveness of the considered classifiers in terms of filtering the recorded signals.

The development of reliable tools for distinguishing signals from various types of noise, allowing precise filtering of the CREDO dataset is essential for further meta-research involving the verification of relevant physical hypotheses, such as the detection of particle bursts, correlations between single bursts, determination of propagation directions of primary particles, and more advanced astrophysical analyses. The procedures developed in the work described by this paper, including verified classifier models are, in our opinion, an important step towards building a robust instrumentarium to increase the potential of CREDO data analysis in this field and to enable physicists to perform more authoritative analyses.

## Figures and Tables

**Figure 10:**
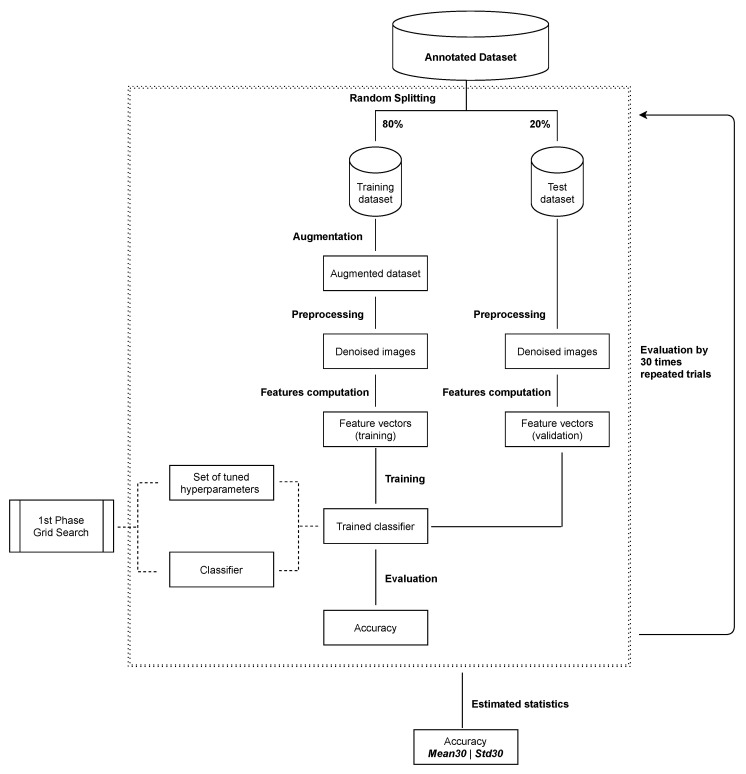
Descriptive scheme for the second phase of computation: model evaluation using 30 repeated classification trials.

**Table 1:** Key metrics related to the CREDO data set as of September 2021.

User base	$1.4\times 10^{4}$
Device base	$1.5\times 10^{4}$
Operation time	$3.9\times 10^{5}$ days (~1050 years)
Candidate detections	$1.0\times 10^{7}$

**Table 2:** Class multiplicities in the annotated data set. The symbol # indicates the cardinality of a subset for a given class, and % indicates the relative quantitative contribution of such a subset to the entire training set.

	Spots	Tracks	Worms	Artefacts
#	535	393	304	1150
%	22.5	16.5	12.8	48.2

**Table 3:** Statistics describing the partitioning and augmentation scheme of the dataset used in the first phase of computation (Figure 9).

	Spots	Tracks	Worms	Artefacts	Total
Data set	535	393	304	1150	2382
Test set	107	79	61	230	477
Optimization set	428	314	243	920	1905
Augmented opt. set	428	2198	3159	920	6705
Augmented training set	342	1758	2527	736	5363
Augmented validation set	86	440	632	184	1342

**Table 4:** Statistics describing the partitioning and augmentation scheme of the dataset used in the second phase of computation (Figure 10).

	Spots	Tracks	Worms	Artefacts	Total
Data set	535	393	304	1150	2382
Test set	107	79	61	230	477
Training set	428	314	243	920	1905
Augmented training set	428	2198	3159	920	6705

**Table 5:** Accuracy of the optimized basic classifiers (1st phase) and the results of their validation (2nd phase). The best results, where each value of CV,Test,Mean30>0.85 are in bold. The scaling procedures are denoted as follows: z-score for standardization and norm for normalization.

	1st Phase				2nd Phase	
Classifier	CV	Test	Scaling	Hyperparameters	Mean30	Std30
DTC	0.8037	0.7966	z-score	{’criterion’: ’entropy’, ’splitter’: ’random’}	0.8194	0.0183
GNB	0.4764	0.7191	z-score	no parameters were optimized	0.7017	0.0216
KNN	0.8415	0.7883	z-score	{’metric’: ’chebyshev’, ’n_neighbors’: 1}	0.7808	0.0188
LDA	0.6534	0.5723	z-score	{’shrinkage’: None, ’solver’: ’lsqr’}	0.5538	0.0240
LRC	0.8847	0.8470	z-score	{’penalty’: ’none’, ’solver’: ’newton-cg’}	0.8292	0.0178
LSV	0.8449	0.8050	z-score	{’C’: 10, ’class_weight’: None}	0.7843	0.0222
**MLP**	**0.9154**	**0.8784**	z-score	{’activation’: ’relu’, ’hidden’: (180, 120), ’solver’: ’adam’}	**0.8799**	0.0134
**NSV**	**0.9016**	**0.9015**	z-score	{’gamma’: 0.1, ’kernel’: ’rbf’, ’nu’: 0.05}	**0.8711**	0.0136
QDA	0.6999	0.7631	z-score	{’reg_param’: 0.0001}	0.7422	0.0209
SGD	0.8683	0.8008	z-score	{’alpha’: 9e-05, ’loss’: ’log’, ’penalty’: ’l1’}	0.8027	0.0202
**SVC**	**0.9177**	**0.8952**	z-score	{’C’: 700, ’gamma’: 0.08, ’kernel’: ’rbf’}	**0.8818**	0.0124

**Table 6:** Accuracy of the optimized ensemble classifiers (1st phase) and the results of their validation (2nd phase). The results are too similar to each other to determine a best result, so no model was chosen. The scaling procedures are denoted as follows: z-score for standardization and norm for normalization.

	1st Phase				2nd Phase	
Classifier	CV	Test	Scaling	Hyperparameters	Mean30	Std30
ETC	0.8986	0.8973	norm	{’bootstrap’: False, ’max_features’: None, ’criterion’: ’gini’, ’n_estimators’: 70}	0.8758	0.0142
GBC	0.8950	0.8847	norm	{’learning_rate’: 0.7, ’n_estimators’: 90 ’max_depth’: 9}	0.8741	0.0150
RFC	0.8853	0.8763	norm	{’bootstrap’: False, ’criterion’: ’entropy’, ’max_features’: 5, ’n_estimators’: 40}	0.8699	0.0137
VOT	0.9205	0.8973	z-score	{’weights’: (4, 8, 8)}	0.8841	0.0123
BAG/SVC	0.9078	0.8868	z-score	{’max_samples’: 0.7, ’n_estimators’: 100}	0.8805	0.0135
OvO/MLP	0.9101	0.8973	z-score	no parameters were optimized	0.8880	0.0145
OvO/SVC	0.9171	0.8889	z-score	no parameters were optimized	0.8850	0.0148
OvR/MLP	0.9138	0.8952	z-score	no parameters were optimized	0.8853	0.0139

**Table 7:** Comparison of final results for each class (spots, tracks, worms, artefacts) in selected methods in different works (publications: Hachaj et al. [32], Winter et al. [13] and our model “MLP with one-vs-rest multiclass strategy” from Figure 11i.

Class	Hachaj et al.	Winter et al.	This Paper
Spots	98.71%	98.9%	98.13%
Tracks	88.89%	95.4%	93.67%
Worms	89.65%	92.9%	88.52%
Artefacts	97.70%	98.2%	91.96%

## Data Availability

The training set as well as the source code used in this analysis are available at https://github.com/credo-ml/feature-based-classifiers, accessed on 17 November 2021.
